# Evaluation of the Atherogenic Index of Plasma in the Prognostic Value of Ischemic Heart Failure Post-Percutaneous Coronary Intervention

**DOI:** 10.31083/RCM33470

**Published:** 2025-06-30

**Authors:** Yinxiao Xu, Biyang Zhang, Meishi Ma, Yi Kan, Tienan Sun, Xin Huang, Yujie Zhou

**Affiliations:** ^1^Department of Cardiology, Capital Medical University Affiliated Anzhen Hospital, 100089 Beijing, China

**Keywords:** atherogenic index of plasma, ischemic heart failure, percutaneous coronary intervention, MACE, prognosis

## Abstract

**Background:**

The atherogenic index of plasma (AIP) is calculated as the logarithm of the triglyceride (TG) to high-density lipoprotein cholesterol (HDL-C) ratio. While previous studies suggested that TG and HDL-C levels were linked to the prognosis in various cardiovascular conditions, including ischemic heart failure (IHF), there is limited research specifically examining AIP in the context of IHF. Therefore, our study sought to explore the association between AIP and the prognosis of IHF and to compare the predictive value of AIP, HDL-C, and TG levels for identifying patients with poor outcomes.

**Methods:**

This retrospective cohort study was conducted at a single institution involving 2036 IHF patients with post-percutaneous coronary intervention (PCI) who were followed for 36 months. Patients were divided into four groups categorized according to AIP quartiles. The primary outcome of interest was major adverse cardiovascular events (MACEs), while secondary outcomes included all-cause mortality, non-fatal myocardial infarction (MI), and any revascularization. Kaplan–Meier survival curves were used to evaluate the occurrence of endpoints across the four groups. Multivariate Cox regression analysis reinforced that AIP independently predicted primary and secondary outcomes. Restricted cubic spline (RCS) method was employed to examine the non-linear association between AIP and endpoints. Receiver operating characteristic (ROC) curves, combined with the Delong test, were used to assess and compare the predictive accuracy of AIP, TG, and HDL-C.

**Result:**

The incidence of MACEs (Q4:Q1 = 50.6:23.0, *p* < 0.001), all-cause death (Q4:Q1 = 25.0:11.6, *p* < 0.001), and any revascularization (Q4:Q1 = 21.6:9.6, *p* < 0.001) were significantly higher in patients with elevated AIP. The Kaplan– Meier curve analysis further supported a positive association between AIP and MACEs (*p*_log-rank_ < 0.001). Multivariate Cox analysis showed that AIP was independently associated with the increased risk of MACEs (Q4:Q1 (HR (95% CI)): 2.84 (2.25–3.59), *p*_trend_ < 0.001), all-cause death (Q4:Q1 (HR (95% CI)): 2.76 (1.98–3.84), *p*_trend_ < 0.001), non-fatal MI (Q4:Q1 (HR (95% CI)): 3.01 (1.32–6.90), *p*_trend_ < 0.001), and any revascularization (Q4:Q1 (HR (95% CI)): 2.92 (2.04–4.19), *p*_trend_ < 0.001). In RCS, higher AIP was non-linearly relevant to an increased risk of MACEs (*p*_non-linear_ = 0.0112). In subgroup analysis, the predictive value of AIP for MACEs was more pronounced in the younger patient subgroup (*p*_interaction_ = 0.003). The ROC curves showed the predictive value of AIP (area under curve [AUC] = 0.641), HDL-C (AUC = 0.600), and TG (AUC = 0.629), and AIP had the best predictive value among TG (AIP:TG: difference in AUC (95% CI), 0.012 (0.001–0.024), *p* for Delong test = 0.028) and HDL-C (AIP:HDL-C: difference in AUC (95% CI), 0.041 (0.018–0.064), *p* for Delong test <0.001).

**Conclusion:**

In IHF patients after PCI, AIP was strongly relevant to an increased risk of MACEs and had the best predictive validity compared with TG and HDL-C.

## 1. Introduction

Over the past decade, global deaths from cardiovascular disease have risen by 12.5%. Cardiovascular diseases (CVD) now contribute to one-third of global fatalities [1]. This increase was largely due to population growth and aging, with the highest mortality rates occurring in South and East Asian countries due to their large and expanding populations [2]. Among patients who died from CVD, coronary artery disease (CAD), a severe narrowing of the coronary arteries caused by a variety of factors, accounted for the highest proportion of fatalities [2, 3]. Furthermore, epidemiological studies indicated that ischemic heart disease (IHF) was the primary cause of heart failure (HF) [4]. CAD, a major subtype of ischemic heart disease, triggers physiological changes that can negatively affect myocardial function, contributing to the development of HF [5]. When CAD was followed by secondary HF, also known as IHF [6], patients generally tended to have a worse prognosis [7]. Therefore, the use of simple and easily available laboratory predictors for the prognosis of IHF and the early identification of high-risk patients would be beneficial in reducing the burden of healthcare on society globally (especially in developing countries). Several previous studies have tested predictors of IHF, such as the systemic inflammation response index, positron emission tomographic metrics, and B-natriuretic peptide (BNP) [8, 9, 10]. Some studies further provided new insights and approaches for identifying high-risk patients and advancing precise medicine through artificial intelligence and other methods [11]. However, some new risk factors need to be investigated, which may lead to new guidelines for early clinical interventions in IHF patients with a poor prognosis.

The atherogenic index of plasma (AIP) is calculated as the logarithm of the triglyceride (TG) to high-density lipoprotein cholesterol (HDL-C) ratio, providing an indication of the balance between these two lipid components [12]. AIP is a reliable marker for assessing the extent of coronary atherosclerosis and has proven to be an effective prognostic tool for various CVDs [13]. Previous studies have also found a high AIP was associated with a poor prognosis in diabetes, renal insufficiency, acute ischemic stroke and other conditions [14, 15, 16]. For patients with IHF, however, the predictive validity of AIP has not been fully elucidated from current studies. TG and HDL-C, as components of AIP, have been found to be predictive of the prognosis of IHF in previous studies [17, 18]. Hypertriglyceridemia accelerates the development of CVD through mechanisms such as promoting atherosclerosis and increasing blood viscosity. Several TG-related indices, such as the triglyceride-glucose index, play a significant role in the occurrence of coronary artery disease, heart failure, and other cardiovascular events [19, 20]. Nevertheless, there were few studies comparing the predictive value of AIP, TG, and HDL-C specifically for IHF post-percutaneous coronary intervention (PCI). We explored how AIP relates to the prognosis of IHF, and further compared the predictive efficacy of AIP with its components (TG and HDL-C).

## 2. Method

### 2.1 Cohort of Participants

**Fig. 1.**
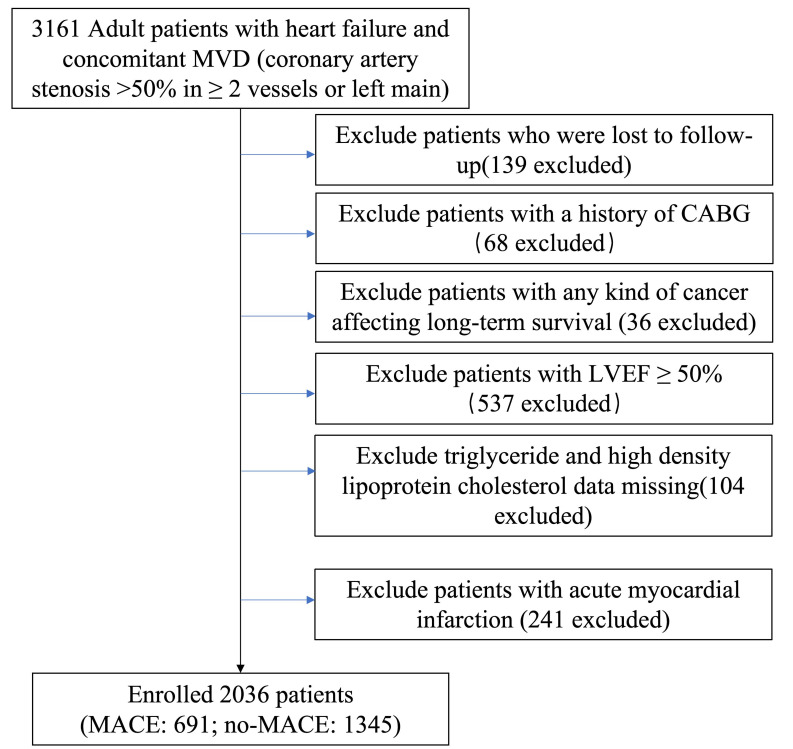
**Diagram of study process**. Abbreviations: MVD, multivessel disease; CABG, coronary artery bypass grafting; LVEF, left ventricular ejection fraction; MACE, major adverse cardiovascular events.

This observational, retrospective cohort study was conducted at the Beijing Anzhen Hospital, recruiting IHF patients who underwent selective PCI during Jun. 2017 to Jun. 2019. IHF was identified from these criteria [6]: (1) HF was diagnosed based on the criteria outlined in the 10th revision of the International Classification of Diseases (ICD-10). This included patients with clinical symptoms such as dyspnea, fatigue, and fluid retention, as well as objective evidence of cardiac dysfunction confirmed by echocardiography or other diagnostic tools. (2) Multivessel disease (MVD), defined as either left main coronary artery involvement or ≥50% stenosis in at least two major coronary arteries, as determined by coronary angiography. This cohort comprised 3161 IHF patients. Exclusion criteria included: (1) Patients who were lost to follow-up, meaning they did not complete the required follow-up visits or had insufficient clinical data. (2) Patients with a left ventricular ejection fraction (LVEF) of ≥50%, as heart failure with preserved ejection fraction (HFpEF) was not the focus of this study. (3) Patients with a recent history of coronary artery bypass grafting (CABG), defined as undergoing the procedure within the past 6 months, since surgical intervention could significantly impact long-term outcomes. (4) Patients diagnosed with acute myocardial infarction (AMI) at the time of admission, as acute ischemic events may have confounded the assessment of chronic ischemic heart failure. (5) Patients with malignant tumors, as cancer-related cachexia and systemic inflammation could influence cardiovascular outcomes. (6) Patients with missing TG or HDL-C data, since these lipid parameters were essential for the study’s analysis. The final analysis included 2036 patients (Fig. 1).

### 2.2 Information Retrieval and Variable Definitions

Data were extracted from the electronic medical records at the Beijing Anzhen Hospital. All laboratory parameters in the baseline table were obtained from the first fasting blood test after admission. The examination results were taken from the first assessment conducted after admission. Medication use information was recorded based on the treatments during hospitalization. AIP was calculated from TG and HDL-C in the baseline. Angiographic images were assessed by a minimum of two experienced cardiologists. The synergy between PCI with taxus and cardiac surgery (SYNTAX) score was calculated using the algorithm available on https://www.syntaxscore.com.

### 2.3 Outcomes 

Following the initial PCI, patients were monitored at 3, 6, 9, 12, 24, 36 months by trained healthcare professionals. Data on outcomes and medication consumption were collected via phone surveys or patient visits to the outpatient clinic. Major adverse cardiovascular events (MACE) included: all-cause mortality, non-fatal myocardial infarction (MI), any revascularization procedure. MI was defined based on the fourth universal definition of MI (2018) [21], while revascularization referred to any coronary revascularization procedure performed for any reason.

### 2.4 Grouping and Endpoints

AI*p* = log(TG/HDL-C). Our cohort was classified into four groups based on AIP levels: Quartile (Q) 1 (AIP ≤0.03), Q2 (0.03 < AIP ≤ 0.40), Q3 (0.40 < AIP ≤ 0.80), Q4 (AIP >0.80). The primary outcome assessed was MACE. Each component of the primary outcome was considered a secondary endpoint. Various adverse events were recorded throughout the follow-up period, but only first-time adverse events were analyzed. The follow-up period extended until Jun. 2022.

### 2.5 Analytical Methods

For normally distributed continuous variables, data were presented as mean ± standard deviation (SD) and analyzed via the ANOVA test. Non-normally distributed continuous variables were described using the median and interquartile range (IQR), with group differences assessed by the Kruskal-Wallis test. Categorical variables were reported as counts (percentages) and compared using the Chi-square test.

Multivariable Cox proportional hazard models were utilized to analyze the independent effect of AIP on endpoints, with results presented as hazard ratios (HR) and 95% confidence intervals (CI). Variables in multivariate regression model II were those that were clinically significant and had between-group differences. Variables in the multivariate regression model III were selected by stepwise regression method of analysis (*p* < 0.05). Q1 of the AIP served as the reference for comparison. Model I was an unadjusted model without any covariates. Model II adjusted age, sex, body mass index, diabetes, red blood cell, platelet, hemoglobin, total cholesterol (TC), glycosylated hemoglobin A1c (HbA1c), chronic total occlusion, left atrial diameter, left ventricular end-systolic diameter (LVDs), left ventricular end-diastolic diameter (LVDd). Model III incorporated age, sex, heart rate, body mass index, New York Heart Association (NYHA) class, prior PCI, white blood cell count, neutrophil count, mononuclear cell count, albumin, creatinine, estimated glomerular filtration rate (eGFR), uric acid, sacubitril valsartan, sulfonylurea, in-stent restenosis, SYNTAX score, and complete revascularization. Kaplan-Meier survival curves were utilized to analyze the occurrence of endpoints across four AIP quartiles. The Restricted cubic spline (RCS) model analyzed non-linearity in AIP to MACE. The number of knots was determined by Akaike information criterion, selecting 4 knots. Subgroup analysis explored the correlation of AIP and primary outcomes in several subgroups. Receiver operating characteristic (ROC) curves along with the Delong test compared predictive values of three indicators. Statistical analyses utilized R software (version 4.2.1, R Foundation for Statistical Computing, Vienna, Austria) and Stata version 18.0 (StataCorp, College Station, TX, USA). A *p*-value of less than 0.05 was regarded as statistically significant.

## 3. Result

### 3.1 Cohort Characteristics 

2036 individuals (362 women and 1674 men) were included. The median (IQR) of the AIP level was 0.40 (0.03–0.80). As the AIP quartiles increased, patients tended to be younger, with a higher proportion of males, elevated heart rates, higher body mass indices, and a greater prevalence of diabetes and a prior history of PCI. The high AIP grouping had higher lymphocyte, red blood cell, platelet, hemoglobin, triglyceride, albumin, TC, uric acid, HbA1c, left atrial diameter, LVDs, LVDd and lower levels of aspartate transaminase (AST), HDL-C, and BNP. Additionally, higher AIP patients were more likely to be on beta-blockers, loop diuretics, metformin, and to have a higher incidence of chronic total occlusion (Table 1).

**Table 1.** **AIP quartile-based stratification of patient characteristics**.

Characteristics			Total (n = 2036)	Quartiles of AIP				*p* value
				Quantile 1 (n = 509)	Quantile 2 (n = 508)	Quantile 3 (n = 515)	Quantile 4 (n = 504)	
				AIP ≤0.03	0.03 < AIP ≤ 0.40	0.40 < AIP ≤ 0.80	AIP >0.80	
Age (years)			60.21 ± 11.02	63.47 ± 9.91	61.59 ± 10.25	58.57 ± 11.24	57.19 ± 11.51	<0.001
Sex, n (%)								0.020
	Male		1674 (82.2)	404 (79.4)	408 (80.3)	444 (86.2)	418 (82.9)	
	Female		362 (17.8)	105 (20.6)	100 (19.7)	71 (13.8)	86 (17.1)	
Vital signs								
	Heart rate (beats/min)		73.76 ± 10.69	72.91 ± 10.20	73.32 ± 10.38	74.00 ± 10.77	74.83 ± 11.30	0.025
	Body mass index (kg/m^2^)		25.72 ± 3.23	24.71 ± 3.09	25.32 ± 3.08	26.12 ± 3.15	26.74 ± 3.23	<0.001
	Systolic blood pressure (mmHg)		122.22 ± 18.11	123.10 ± 18.77	121.94 ± 18.75	121.83 ± 17.21	122.01 ± 17.70	0.652
	Diastolic blood pressure (mmHg)		73.55 ± 11.54	73.08 ± 11.85	72.80 ± 11.61	73.72 ± 11.37	74.60 ± 11.28	0.062
NYHA class, n (%)								0.136
	I		225 (11.1)	43 (8.4)	63 (12.4)	63 (12.2)	56 (11.1)	
	II		1066 (52.4)	286 (56.2)	248 (48.8)	267 (51.8)	265 (52.6)	
	III		672 (33.0)	164 (32.2)	181 (35.6)	170 (33.0)	157 (31.2)	
	IV		73 (3.6)	16 (3.1)	16 (3.1)	15 (2.9)	26 (5.2)	
Comorbidities, n (%)								
	Atrial fibrillation		85 (4.2)	28 (5.5)	23 (4.5)	19 (3.7)	15 (3.0)	0.211
	Hypertension		1175 (57.7)	284 (55.8)	276 (54.3)	308 (59.8)	307 (60.9)	0.103
	Diabetes		786 (38.6)	168 (33.0)	192 (37.8)	191 (37.1)	235 (46.6)	<0.001
	Hypercholesterolemia		1490 (73.2)	386 (75.8)	360 (70.9)	376 (73.0)	368 (73.0)	0.358
History, n (%)								
	Prior stroke		174 (8.5)	57 (11.2)	44 (8.7)	40 (7.8)	33 (6.5)	0.056
	Prior MI		504 (24.8)	113 (22.2)	119 (23.4)	134 (26.0)	138 (27.4)	0.206
	Prior PCI		228 (11.2)	45 (8.8)	69 (13.6)	70 (13.6)	44 (8.7)	0.008
Laboratory parameters								
	White blood cell (10^9^/L)		7.87 ± 2.50	7.79 ± 2.70	7.97 ± 2.68	7.75 ± 2.24	7.98 ± 2.33	0.324
	Neutrophil (10^9^/L)		5.42 ± 2.31	5.49 ± 2.54	5.52 ± 2.50	5.25 ± 2.09	5.40 ± 2.06	0.237
	Mononuclear cell (10^9^/L)		0.48 ± 0.20	0.49 ± 0.22	0.48 ± 0.20	0.47 ± 0.18	0.46 ± 0.18	0.242
	Lymphocyte (10^9^/L)		1.79 ± 0.61	1.64 ± 0.59	1.77 ± 0.59	1.83 ± 0.61	1.92 ± 0.63	<0.001
	Red blood cell (10^9^/L)		4.51 ± 0.56	4.43 ± 0.57	4.46 ± 0.52	4.56 ± 0.56	4.61 ± 0.57	<0.001
	Platelet (10^9^/L)		221.39 ± 60.05	213.28 ± 61.01	220.94 ± 57.44	226.45 ± 60.66	224.89 ± 60.32	0.002
	Hemoglobin (g/L)		138.60 ± 17.06	136.12 ± 16.80	137.38 ± 17.29	139.97 ± 16.73	140.92 ± 17.04	<0.001
	FBG (mmol/L)		3.53 ± 0.94	3.49 ± 1.02	3.50 ± 0.85	3.57 ± 0.93	3.54 ± 0.97	0.565
	AST (U/L)		22 (17, 36)	24 (18, 45)	22 (17, 38)	22 (17, 32)	22 (17, 32)	0.003
	ALT (U/L)		25 (16, 40)	25 (16, 43)	24 (15, 39)	25 (17, 39)	25 (17, 40)	0.204
	Albumin (g/L)		41.76 ± 3.88	41.37 ± 3.96	41.42 ± 3.86	41.93 ± 3.90	42.34 ± 3.75	<0.001
	eGFR (mL/min/1.73 m^2^)		87.59 ± 21.06	87.97 ± 19.61	86.08 ± 20.69	88.42 ± 21.85	87.87 ± 21.99	0.300
	Triglyceride (mmol/L)		1.69 ± 1.04	0.89 ± 0.22	1.29 ± 0.27	1.67 ± 0.34	2.95 ± 1.33	<0.001
	TC (mmol/L)		4.07 ± 1.09	4.06 ± 1.11	3.98 ± 1.01	4.00 ± 1.03	4.25 ± 1.19	<0.001
	HDL-C (mmol/L)		1.01 ± 0.25	1.23 ± 0.24	1.03 ± 0.21	0.92 ± 0.17	0.84 ± 0.17	<0.001
	LDL-C (mmol/L)		2.43 ± 0.90	2.42 ± 0.96	2.45 ± 0.88	2.45 ± 0.85	2.41 ± 0.93	0.845
	Uric acid (µmol/L)		369.34 ± 101.82	335.10 ± 93.63	365.68 ± 98.98	376.55 ± 98.64	400.25 ± 105.18	<0.001
	Sodium (mmol/L)		139.01 ± 3.02	138.85 ± 3.17	139.11 ± 3.05	138.98 ± 3.09	139.09 ± 2.77	0.500
	Potassium (mmol/L)		4.16 ± 0.44	4.15 ± 0.44	4.16 ± 0.45	4.18 ± 0.44	4.15 ± 0.41	0.643
	HbA1c (%)		6.80 ± 1.39	6.62 ± 1.39	6.68 ± 1.33	6.86 ± 1.41	7.02 ± 1.42	<0.001
	BNP (pg/mL)		331 (144, 477)	363 (178, 512)	334 (146, 491)	310 (150, 458)	302 (115, 459)	0.002
	hs-CRP (mg/L)		6.68 ± 11.04	7.29 ± 13.62	6.78 ± 10.54	6.48 ± 10.22	6.18 ± 9.27	0.421
	AIP		0.40 (0.03, 0.80)	–0.28 (–0.51, –0.12)	0.23 (0.13, 0.32)	0.58 (0.48, 0.68)	1.09 (0.92, 1.38)	<0.001
Echocardiography								
	Left atrial diameter (millimeter)		39.28 ± 5.17	38.56 ± 5.41	39.39 ± 5.04	39.59 ± 5.13	39.57 ± 5.03	0.003
	LVDs (millimeter)		41.20 ± 7.97	40.25 ± 7.91	41.04 ± 7.79	41.77 ± 8.17	41.73 ± 7.91	0.006
	LVDd (millimeter)		54.83 ± 7.12	53.96 ± 7.08	54.55 ± 7.14	55.32 ± 7.11	55.51 ± 7.07	0.002
	LVEF (%)		41.08 ± 6.01	41.14 ± 5.87	41.11 ± 6.18	40.96 ± 6.29	41.12 ± 5.70	0.963
Medication use, n (%)								
	Aspirin		2029 (99.7)	508 (99.8)	507 (99.8)	513 (99.6)	501 (99.4)	0.659
	Clopidogrel		1636 (80.4)	417 (81.9)	423 (83.3)	390 (75.7)	406 (80.6)	0.015
	Ticagrelor		399 (19.6)	92 (18.1)	84 (16.5)	125 (24.3)	98 (19.4)	0.012
	Statins		2024 (99.4)	507 (99.6)	503 (99.0)	514 (99.8)	500 (99.2)	0.331
	Ezetimibe		500 (24.6)	126 (24.8)	125 (24.6)	105 (20.4)	144 (28.6)	0.026
	Oral anticoagulants		91 (4.5)	23 (4.5)	23 (4.5)	23 (4.5)	22 (4.4)	0.999
	Warfarin		38 (1.9)	9 (1.8)	9 (1.8)	12 (2.3)	8 (1.6)	0.833
	Xa inhibitors		33 (1.6)	11 (2.2)	9 (1.8)	5 (1.0)	8 (1.6)	0.499
	IIa inhibitors		20 (1.0)	3 (0.6)	5 (1.0)	6 (1.2)	6 (1.2)	0.751
	CCB		258 (12.7)	62 (12.2)	61 (12.0)	76 (14.8)	59 (11.7)	0.430
	Beta-blockers		1225 (60.2)	265 (52.1)	297 (58.5)	330 (64.1)	333 (66.1)	<0.001
	ACEI		172 (8.4)	36 (7.1)	55 (10.8)	42 (8.2)	39 (7.7)	0.148
	ARB		234 (11.5)	49 (9.6)	57 (11.2)	68 (13.2)	60 (11.9)	0.341
	Diuretics		1353 (66.5)	320 (62.9)	333 (65.6)	347 (67.4)	353 (70.0)	0.101
	Loop diuretics		1165 (57.2)	267 (52.5)	286 (56.3)	300 (58.3)	312 (61.9)	0.022
	Thiazine diuretics		102 (5.0)	22 (4.3)	25 (4.9)	21 (4.1)	34 (6.7)	0.200
	Spironolactone		944 (46.4)	236 (46.4)	241 (47.4)	237 (46.0)	230 (45.6)	0.947
	Sacubitril valsartan		692 (34.0)	167 (32.8)	170 (33.5)	166 (32.2)	189 (37.5)	0.278
	Tovaputan		64 (3.1)	24 (4.7)	14 (2.8)	14 (2.7)	12 (2.4)	0.130
	Metformin		195 (9.6)	31 (6.1)	51 (10.0)	49 (9.5)	64 (12.7)	0.005
	Sulfonylurea		43 (2.1)	11 (2.2)	11 (2.2)	12 (2.3)	9 (1.8)	0.942
	Insulin		472 (23.2)	107 (21.0)	116 (22.8)	127 (24.7)	122 (24.2)	0.513
Angiographic data								
	Chronic total occlusion, n (%)		556 (27.3)	124 (24.4)	121 (23.8)	158 (30.7)	153 (30.4)	0.014
	Three‑vessel disease, n (%)		1161 (57.0)	275 (54.0)	285 (56.1)	308 (59.8)	293 (58.1)	0.804
	LM disease, n (%)		367 (18.0)	94 (18.5)	94 (18.5)	95 (18.4)	84 (16.7)	0.840
	Diffuse lesion, n (%)		389 (19.1)	108 (21.2)	85 (16.7)	96 (18.6)	100 (19.8)	0.312
	In-stent restenosis, n (%)		88 (4.3)	25 (4.9)	25 (4.9)	24 (4.7)	14 (2.8)	0.270
	SYNTAX score		21.9 ± 7.7	21.6 ± 7.6	21.4 ± 7.3	22.2 ± 8.3	22.1 ± 7.7	0.264
Procedural results								
	Target vessel territory, n (%)							
		LM	334 (16.4)	84 (16.5)	86 (16.9)	87 (16.9)	77 (15.3)	0.882
		LAD	1542 (75.7)	396 (77.8)	390 (76.8)	381 (74.0)	375 (74.4)	0.419
		LCX	1305 (64.1)	313 (61.5)	322 (63.4)	342 (66.4)	328 (65.1)	0.389
		RCA	1409 (69.2)	345 (67.8)	358 (70.5)	356 (69.1)	350 (69.4)	0.830
	Complete revascularization, n (%)		1240 (60.9)	314 (61.7)	317 (62.4)	304 (59.0)	305 (60.5)	0.705
	Number of stents		3.3 ± 1.5	3.3 ± 1.6	3.4 ± 1.5	3.3 ± 1.4	3.4 ± 1.5	0.722

Abbreviation: AIP, atherogenic index of plasma; NYHA, New York Heart Association; MI, myocardial infarction; PCI, percutaneous coronary intervention; FBG, fasting blood glucose; AST, aspartate transaminase; ALT, alanine transaminase; eGFR, estimated glomerular filtration rate; TC, total cholesterol; LDL-C, low-density lipoprotein cholesterol; HDL-C, high-density lipoprotein cholesterol; HbA1c, glycosylated hemoglobin A1c; BNP, B-natriuretic peptide; hs-CRP, high sensitivity C-reactive protein; LVDs, left ventricular end-systolic diameter; LVDd, left ventricular end-diastolic diameter; CCB, calcium channel blocker; ACEI, angiotensin-converting enzyme inhibitor; ARB, angiotensin receptor blocker; LM, left main artery; LAD, left anterior descending artery; LCX, left circumflex artery; RCA, right coronary artery; SYNTAX, synergy between PCI with taxus and cardiac surgery.

### 3.2 Association Between AIP and MACE

During the follow-up period, 691 (33.9%) primary endpoint events were recorded totally, as detailed in Table 2. These included 330 (16.2%) cases of all-cause mortality, 70 (3.4%) cases of non-fatal MI, and 291 (14.3%) cases of any revascularization. The data indicated a significant increase in the incidence of MACE (*p* < 0.001), all-cause mortality (*p* < 0.001), and any revascularization (*p* < 0.001) with rising AIP levels. None of the significant differences in the rate of non-fatal MI, were observed (*p* = 0.095) across the four groups (Table 2).

**Table 2.** **Outcomes across different AIP quartiles**.

Outcomes	Total (n = 2036)	Quartiles of AIP				*p* value
		Quantile 1 (n = 509)	Quantile 2 (n = 508)	Quantile 3 (n = 515)	Quantile 4 (n = 504)	
		AIP ≤0.03	0.03 < AIP ≤ 0.40	0.40 < AIP ≤ 0.80	AIP >0.80	
MACE, n (%)	691 (33.9)	117 (23.0)	125 (24.6)	194 (37.7)	255 (50.6)	<0.001
All-cause mortality	330 (16.2)	59 (11.6)	57 (11.2)	88 (17.1)	126 (25.0)	<0.001
Non-fatal MI	70 (3.4)	9 (1.8)	18 (3.5)	23 (4.5)	20 (4.0)	0.095
Any revascularization	291 (14.3)	49 (9.6)	50 (9.8)	83 (16.1)	109 (21.6)	<0.001

**Fig. 2.**
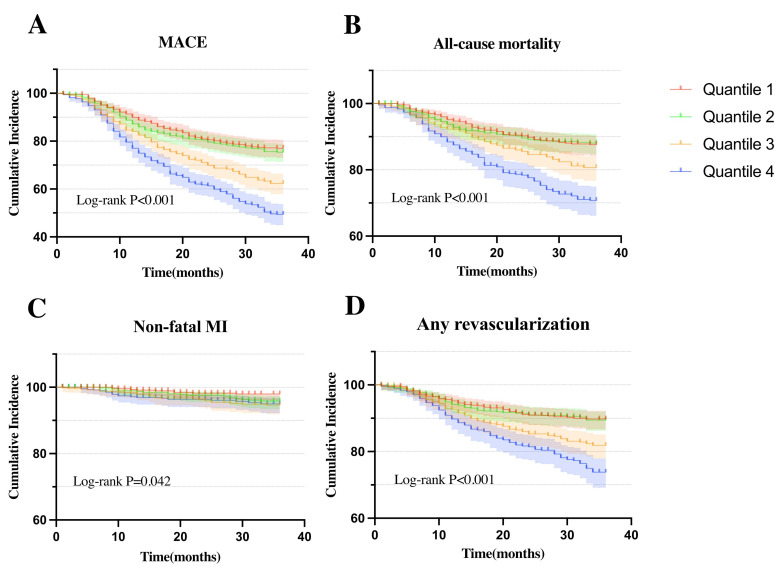
**Kaplan-Meier curves for cumulative incidence of clinical outcomes stratified by quartiles**. (A) Kaplan-Meier curves of MACE. (B) Kaplan-Meier curves of all-cause mortality. (C) Kaplan-Meier curves of non-fatal MI. (D) Kaplan-Meier curves of any revascularization.

Fig. 2 illustrated Kaplan-Meier curves for MACE its components, each categorized by AIP quartiles. The curves show that high AIP group experienced a significantly greater incidence of MACE, all-cause mortality, non-fatal MI, any revascularization compared to low AIP groups (*p*_log-rank_ all <0.05).

The Cox regression was employed to determine the independent impact of AIP on the study outcomes. In Model I, without adjusting for any variables, an increased quartile reflected higher risks of all outcomes. Model II included clinically significant confounding variables and still produced similar results consistent with Model I: MACE (Q4:Q1 [HR, 95% CI] = 2.47 [1.97–3.11], *p* < 0.001, *p*_trend_ < 0.001), all-cause mortality (Q4:Q1 [HR, 95% CI] = 2.47 [1.97–3.11], *p* < 0.001, *p*_trend_ < 0.001), non-fatal MI (Q4:Q1 [HR, 95% CI] = 2.51 [1.11–5.66], *p* = 0.027, *p*_trend_ = 0.003), any revascularization (Q4:Q1 [HR, 95% CI] = 2.67 [1.87–3.79], *p* < 0.001, *p*_trend_ < 0.001). Model III, which included a broader range of potential confounding variables, still showed that higher AIP quartiles were independently linked to an increased risk of MACE (Q4:Q1 [HR, 95% CI] = 2.84 [2.25–3.59], *p* < 0.001, *p*_trend_ < 0.001), all-cause mortality (Q4:Q1 [HR, 95% CI] = 2.76 [1.98–3.84], *p* < 0.001, *p*_trend_ < 0.001), non-fatal MI (Q4:Q1 [HR, 95% CI] = 3.01 [1.32–6.90], *p* = 0.009, *p*_trend_ < 0.001), any revascularization (Q4:Q1 [HR, 95% CI] = 2.92 [2.04–4.19], *p* < 0.001, *p*_trend_ < 0.001). In Model II and III, treating AIP as a continuous variable in the multivariate analysis revealed that each increased AIP unit had a notable impact on higher risks of all outcomes (Table 3).

**Table 3.** **The association between AIP and MACE**.

		Model I			Model II			Model III		
		HR (95% CIs)	*p*	*p* for trend	HR (95% CIs)	*p*	*p* for trend	HR (95% CIs)	*p*	*p* for trend
MACE				<0.001			<0.001			<0.001
	Quantile 1 (n = 509)	1.0 (Ref)			1.0 (Ref)			1.0 (Ref)		
	AIP ≤0.03									
	Quantile 2 (n = 508)	1.09 (0.85–1.40)	0.511		1.12 (0.87–1.44)	0.390		1.10 (0.85–1.43)	0.447	
	0.03 < AIP ≤ 0.40									
	Quantile 3 (n = 515)	1.79 (1.42–2.25)	<0.001		1.72 (1.36–2.17)	<0.001		1.75 (1.38–2.22)	<0.001	
	0.40 < AIP ≤ 0.80									
	Quantile 4 (n = 504)	2.61 (2.10–3.25)	<0.001		2.47 (1.97–3.11)	<0.001		2.84 (2.25–3.59)	<0.001	
	AIP >0.80									
	Continuous	1.86 (1.66–2.09)	<0.001		1.79 (1.58–2.03)	<0.001		1.88 (1.67–2.12)	<0.001	
All-cause mortality				<0.001			<0.001			<0.001
	Quantile 1 (n = 509)	1.0 (Ref)			1.0 (Ref)			1.0 (Ref)		
	AIP ≤0.03									
	Quantile 2 (n = 508)	0.98 (0.68–1.42)	0.932		1.00 (0.69–1.44)	0.982		1.01 (0.70–1.46)	0.965	
	0.03 < AIP ≤ 0.40									
	Quantile 3 (n = 515)	1.61 (1.16–2.24)	0.005		1.49 (1.06–2.08)	0.021		1.53 (1.09–2.15)	0.014	
	0.40 < AIP ≤ 0.80									
	Quantile 4 (n = 504)	2.56 (1.88–3.49)	<0.001		2.31 (1.67–3.19)	<0.001		2.76 (1.98–3.84)	<0.001	
	AIP >0.80									
	Continuous	1.92 (1.62–2.27)	<0.001		1.81 (1.51–2.16)	<0.001		1.94 (1.63–2.31)	<0.001	
Non-fatal MI				0.027			0.003			<0.001
	Quantile 1 (n = 509)	1.0 (Ref)			1.0 (Ref)			1.0 (Ref)		
	AIP ≤0.03									
	Quantile 2 (n = 508)	2.04 (0.91–4.53)	0.081		2.07 (0.93–4.62)	0.077		2.03 (0.90–4.58)	0.090	
	0.03 < AIP ≤ 0.40									
	Quantile 3 (n = 515)	2.76 (1.28–5.97)	0.010		2.76 (1.26–6.05)	0.011		2.62 (1.18–5.79)	0.018	
	0.40 < AIP ≤ 0.80									
	Quantile 4 (n = 504)	2.68 (1.22–5.90)	0.014		2.51 (1.11–5.66)	0.027		3.01 (1.32–6.90)	0.009	
	AIP >0.80									
	Continuous	1.82 (1.26–2.62)	0.001		1.72 (1.17–2.52)	0.006		1.88 (1.28–2.75)	0.001	
Any revascularization				<0.001			<0.001			<0.001
	Quantile 1 (n = 509)	1.0 (Ref)			1.0 (Ref)			1.0 (Ref)		
	AIP ≤0.03									
	Quantile 2 (n = 508)	1.04 (0.70–1.54)	0.848		1.09 (0.73–1.62)	0.666		1.03 (0.69–1.54)	0.872	
	0.03 < AIP ≤ 0.40									
	Quantile 3 (n = 515)	1.82 (1.28–2.60)	0.001		1.82 (1.27–2.60)	0.001		1.87 (1.30–2.68)	0.001	
	0.40 < AIP ≤ 0.80									
	Quantile 4 (n = 504)	2.66 (1.90–3.73)	<0.001		2.67 (1.87–3.79)	<0.001		2.92 (2.04–4.19)	<0.001	
	AIP >0.80									
	Continuous	1.80 (1.50–2.15)	<0.001		1.79 (1.48–2.17)	<0.001		1.83 (1.51–2.20)	<0.001	

**Fig. 3.**
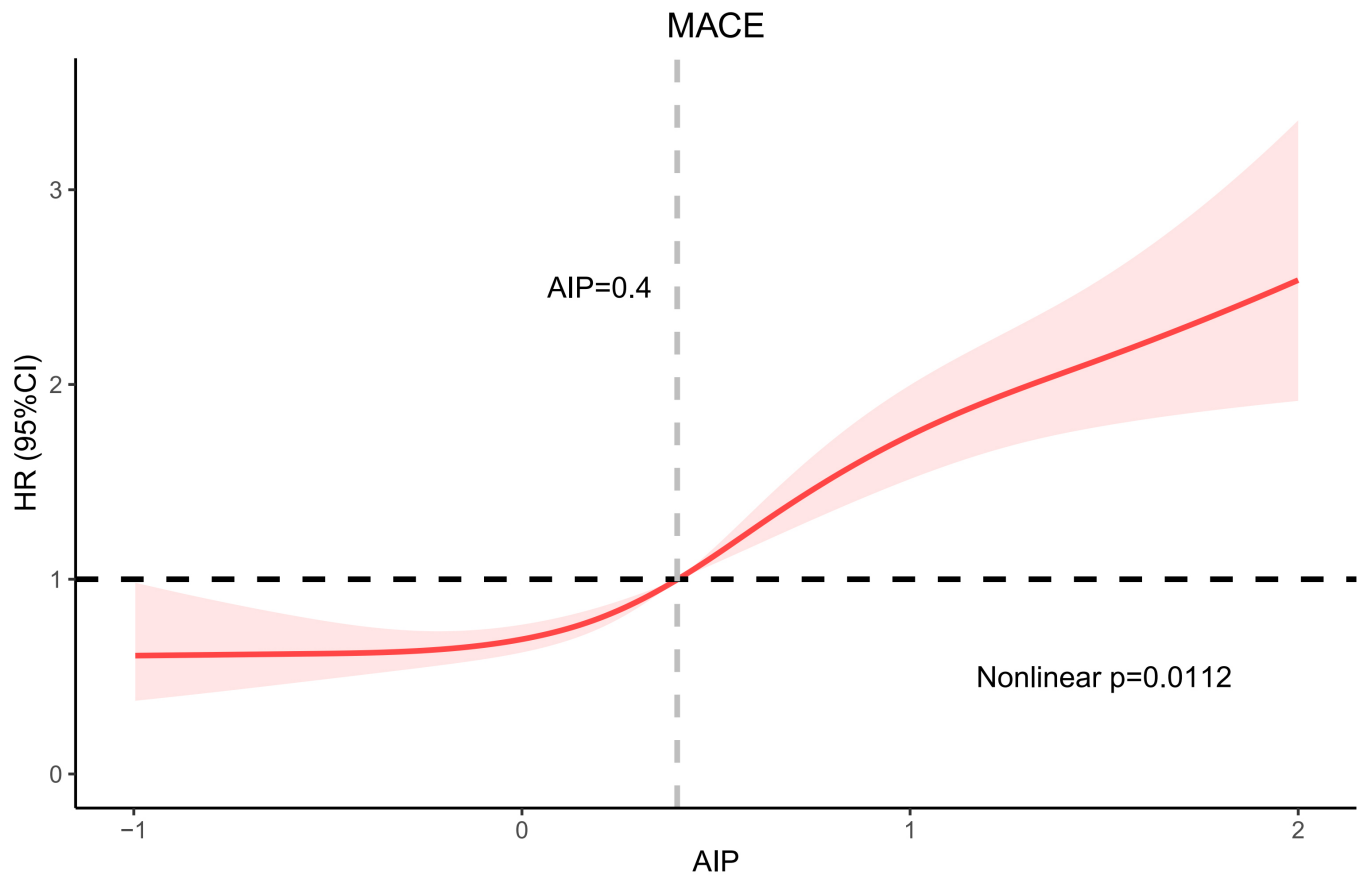
**RCS curve**. Abbreviation: RCS, restricted cubic spline; HR, hazards ratio; CI, confidence interval.

In Fig. 3, RCS was applied to investigate the relationship between AIP and MACE: a high AIP value was non-linearly relevant to increased MACE risk (non-linear, *p* = 0.0112).

**Fig. 4.**
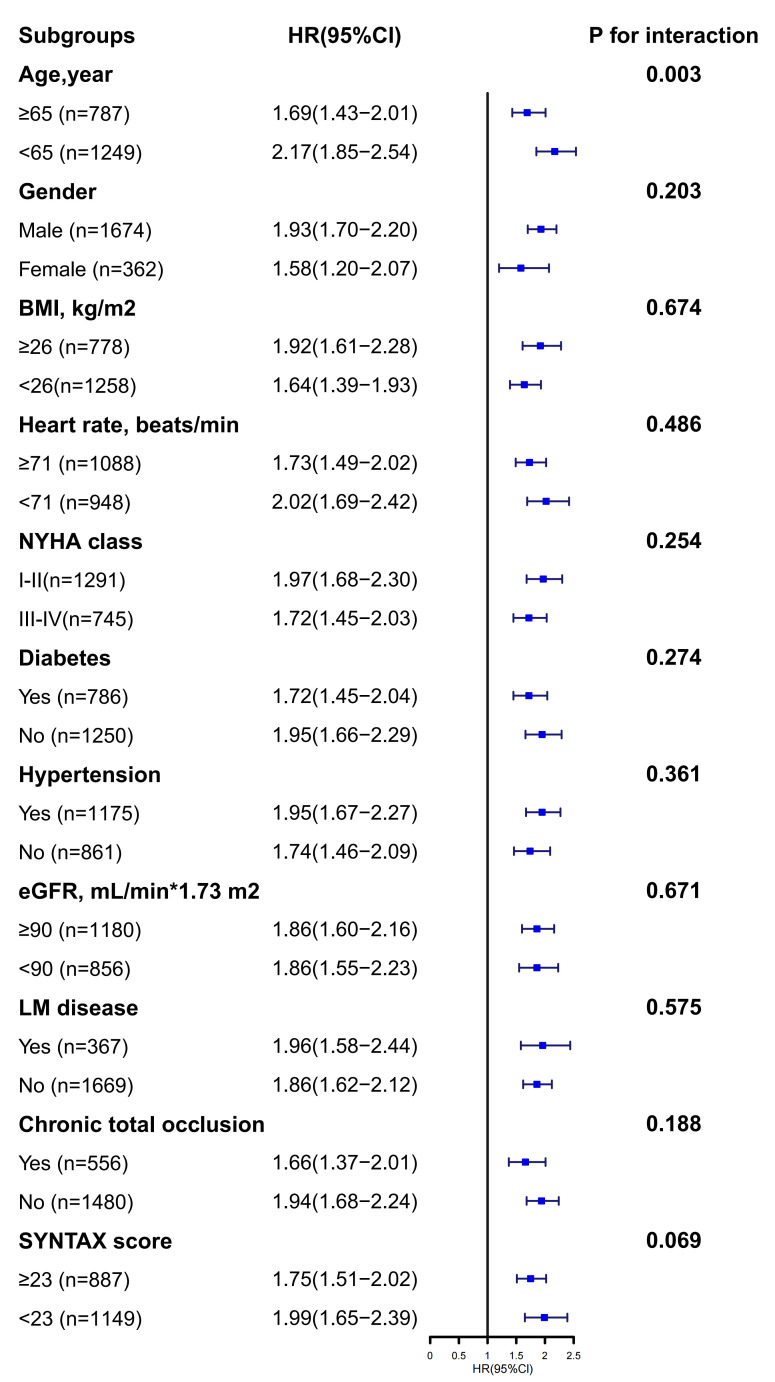
**Subgroup analysis**.

In Fig. 4, significant interactions were not detected in the majority of subgroups, except for the age subgroup. The predictive value of AIP for MACE was notably stronger in the younger patient subgroup (*p*_interaction_ = 0.003).

### 3.3 Comparison of Predictive Value 

**Fig. 5.**
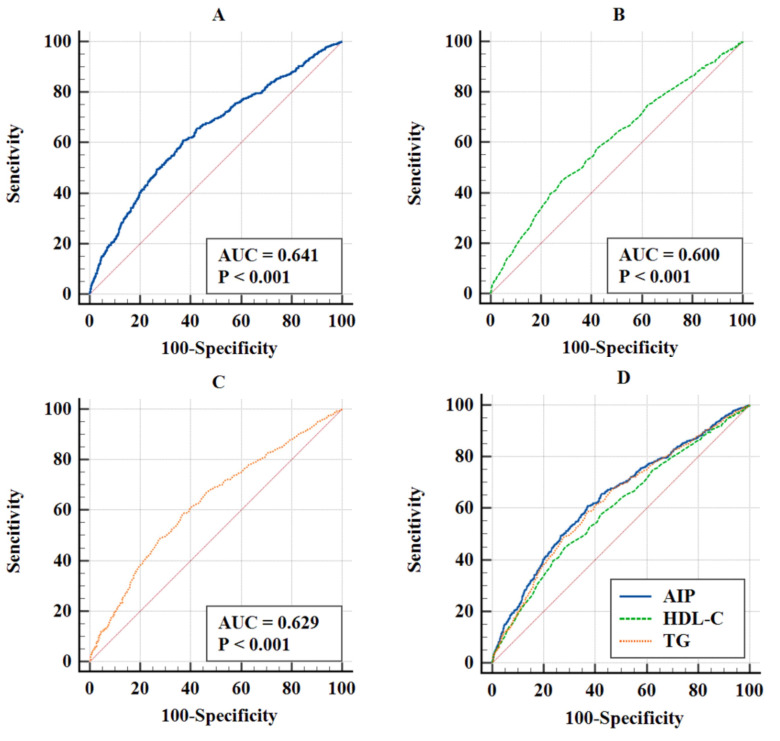
**ROC curves for predicting MACE**. (A) ROC curves of AIP. (B) ROC curves of HDL-C. (C) ROC curves of TG. (D) ROC curves of AIP, HDL-C, TG to MACE. Abbreviation: ROC, receiver operating characteristic.

In Fig. 5, the ROC curves showed the predictive value of AIP (AUC = 0.641, *p* < 0.001), HDL-C (AUC = 0.600, *p* < 0.001), and TG (AUC = 0.629, *p* < 0.001). According to Delong test, AIP had the best predictive value among TG (AIP:TG: difference in AUC (95% CI), 0.012 (0.001–0.024), *p* for Delong test = 0.028) and HDL-C (AIP:HDL-C: difference in AUC (95% CI), 0.041 (0.018–0.064), *p* for Delong test <0.001) (Table 4).

**Table 4.** **Each predictive value at screening for MACE**.

	AUC	95% CI	*p* value	J-index	Optimal cutoff value	Specificity	Sensitivity	Difference in AUC	95% CI	*p* value for Delong test
AIP	0.641	0.620–0.662	<0.001	0.238	0.466	62.9	60.9	Ref	Ref	Ref
HDL-C	0.600	0.579–0.622	<0.001	0.167	0.880	71.8	44.9	0.041	0.018–0.064	<0.001
TG	0.629	0.607–0.650	<0.001	0.215	1.523	63.2	58.3	0.012	0.001–0.024	0.028

Abbreviation: AUC, area under the curve; J-index, Youden index; TG, triglyceride.

## 4. Discussion

Our retrospective study revealed a significant link between AIP and MACE in IHF patients undergoing PCI. As AIP increased, so did the MACE and its components. Kaplan-Meier analysis showed that higher AIP had more frequent adverse events. Even after accounting for potential confounders, higher AIP was consistently and independently related to an augmented risk of these outcomes. RCS analysis confirmed a positive relationship between AIP and MACE. Subgroup analysis revealed a notable interaction effect in the age group. ROC illustrated AIP was the best predictive validity for MACE, followed by TG and HDL-C, respectively.

Dyslipidemia is integral to the development and progression of CVD. Impaired TG metabolism can result in the formation of triglyceride-rich lipoproteins (TRL), which contribute to atherogenesis through various mechanisms [22]. Previous studies have found a causal relationship between elevated TRL and inflammation [23]. This may be because TRLs and their remnants elevate plasma levels of cellular adhesion molecules, which in turn promote the binding of leukocytes and monocytes to atherosclerotic plaques, thereby initiating an inflammatory cascade [24, 25]. Moreover, the buildup of TRLs in plasma contributes to hyper-viscosity by inhibiting fibrinolysis and intensifies the coagulation cascade through increased platelet aggregation, thrombosis, and elevated expression of plasminogen activator inhibitor-1, creating a pro-coagulant environment [25, 26]. Conversely, HDL facilitates the removal of cholesterol out of plaque macrophage foam cells and transports to liver for metabolism and excretion into bile [27, 28]. HDL also exerts anti-inflammatory and antioxidant effects, regulates vascular endothelial homeostasis, and resists thrombosis by reducing platelet aggregation and adhesion reactions [28, 29, 30].

In view of the involvement of TG and HDL-C in atherosclerosis, numerous studies have established a link between AIP and the incidence of cardiovascular disease. A study by Mahdavi-Roshan M *et al*. [31] found that AIP could effectively predict the risk of CAD. Furthermore, higher AIP levels have been observed in HF patients compared to the general population, regardless of the underlying etiology [32]. In contrast, a study from the National Health and Nutrition Examination Survey (NHANES) database found the opposite result. A recent study identified an inverse relationship between AIP and the incidence of HF in a population of 5598 individuals [33]. Therefore, the specific role of AIP in the prognosis in IHF populations requires further exploration. Previous studies have highlighted the predictive value of TG and HDL-C in determining the prognosis of IHF [17, 18], but there was still an absence of available data on the predictive validity of AIP, TG, and HDL-C. We hypothesized that AIP may be a better prognostic predictor for IHF, since it integrates HDL-C and TG in this specific population.

Our study showed that higher AIP was followed by a higher MACE risk. This study also used RCS curves to characterize the relationship between AIP and prognosis in IHF, which was rarely used in other studies of AIP. The RCS curves indicated that the risk of MACE tended to increase as AIP increased, and the slopes of the curves were steepest when AIP was approximately 0.4. Therefore, an AIP value of 0.4 might be a critical threshold that needs to be emphasized, as MACE may increase substantially beyond this point. In the subgroup analyses, no subgroups were identified that would influence the effect of AIP on MACE, except for the age subgroup, which indicated that AIP was a stronger predictor of MACE at ages younger than 65 years. This may be due to the fact that fewer comorbidities and milder conditions in the younger age group reduced the influence of confounding factors on outcomes, thus amplifying the predictive effect of AIP [34]. Therefore, for people younger than 65 years, we should pay more attention to AIP to identify high-risk patients in the clinic, which may result in a higher clinical benefit. Using ROC curves and Delong analysis, we validated the predictive efficacy of AIP in IHF patients, and found that it was superior to TG and HDL-C, two traditional cardiovascular predictors. Therefore, AIP may be useful as a more accurate indicator to be included in prediction models to identify high-risk patients in subsequent studies. In addition, we would also recommend the widespread clinical use of this metric, especially when TG and HDL-C had opposite predictive outcomes in an individual patient.

In previous studies, IHF the population has been characterized by more comorbidities, and a poorer prognosis. After PCI, the prognosis of this population has greater variability. Our study verified a significant positive relationship of AIP and MACE, as confirmed through various analytical models after accounting for potential confounders. We further evaluated the predictive validity (the traditional indices TG and HDL-C versus AIP), and showed that AIP had better predictive validity than TG and HDL-C. Therefore, without adding any additional economic cost in clinical practice, a simple mathematical algorithm can be used to obtain a better prognostic predictor for patients with IHF undergoing PCI. Since South and East Asian countries have the highest proportion of cardiovascular deaths, the use of AIP may be able to improve the cardiovascular mortality rate in economically disadvantaged areas without increasing the economic burden, and even further reduce some of the unnecessary healthcare costs.

## 5. Limitations

(1) The study was retrospective, and patients’ prior treatment may have affected the index measurements. (2) It was a single-center study from the Beijing Anzhen Hospital, one of the largest cardiovascular centers in China, which enrolled patients with predominantly cardiovascular diseases, which may have biased the results and affected the generalizability of the conclusions. (3) We only included fasting laboratory results obtained on the morning following admission and did not conduct dynamic measurements of TG and HDL-C throughout patients’ hospital stay. (4) The study population consisted almost exclusively of Chinese individuals, and it remains unknown whether the findings will vary according to ethnicity. (5) There were limitations in the follow-up methodology of this study that made it difficult to perform dynamic tracking of AIP during the follow-up period.

Prospective studies will be needed to exclude some treatment-induced bias affecting TG and HDL-C. Additionally, we will conduct studies in multiple centers in multiple countries, and a uniform assay will be developed to exclude measurement bias caused by multiple centers while obtaining universal conclusions. In addition, trends in metrics during the patient’s hospitalization will be included to improve the accuracy of the study. Additional research is needed to determine the applicability of the conclusions of this study to the general public. In addition, further improvements will be made in follow-up methods to dynamically assess changes in indicators and outcomes.

## 6. Conclusions

In IHF patients undergoing PCI, higher AIP were linked to an increased risk of MACE, establishing AIP as a moderately predictive prognostic indicator.Additionally, AIP demonstrated superior predictive validity compared to conventional TG and HDL-C.

## Availability of Data and Materials

The datasets used and/or analyzed during the current study are available from the corresponding author upon reasonable request.
